# Staying Alert? Neural Correlates of the Association Between Grit and Attention Networks

**DOI:** 10.3389/fpsyg.2018.01377

**Published:** 2018-08-03

**Authors:** Vrinda Kalia, Robin Thomas, Kira Osowski, Anthony Drew

**Affiliations:** Department of Psychology, Miami University, Oxford, OH, United States

**Keywords:** motivation and attention, grit, perseverance, Attention Network Test, neural correlates

## Abstract

Recent research has demonstrated that heightened motivational levels promote enhanced attention capabilities. However, the relation between attentional systems and the trait-based ability to sustain a motivational state long-term is less understood. Grit refers to one’s ability and willingness to pursue long-term goals despite setbacks. This report presents the results of two studies conducted to examine the relation between facets of Grit–Consistency and Perseverance and attention networks, assessed using the Attention Network Test (ANT). Across both studies Grit–Perseverance was related to performance on the ANT. In Study 1, Grit–Perseverance was negatively related to alerting indicating that individuals who were high on Perseverance were more likely to show a smaller alerting effect. In particular, Grit–Perseverance was negatively correlated with reaction times in the no cue trials. In Study 2, we assessed ERP components associated with attention networks. Individuals with higher scores on Grit–Perseverance were more likely to demonstrate smaller mean difference in N1 amplitudes for double cue relative to no cue trials, suggesting an attenuated *alerting effect*. Our findings indicate that individuals high on Grit–Perseverance may have enhanced sustained attention. Specifically individuals with high Grit–Perseverance appear to exhibit a more efficient alerting system in the no cue trials. Implications of high levels of Grit on cognitive performance are discussed.

## Introduction

It’s a commonly held assumption that perseverance may play a key role in determining individual differences in performance. Sometimes conceptualized as a form of hardiness (Maddi et al., 2012) or courage, perseverance exemplifies an individual’s ability to push through failure and persist with a task (Lucas et al., 2015). In recent years, Duckworth et al. (2007) and Duckworth and Quinn (2009) have encapsulated long-term perseverance toward a valued goal into a personality trait – grit. The psychological literature demonstrates that grit, or “perseverance and passion for long-term goals,” is associated with success in a variety of domains (Duckworth et al., 2007; Duckworth and Eskreis-Winkler, 2013; Rhodes et al., 2017). Grit predicts academic achievement (Duckworth et al., 2007, 2011; Rimfield et al., 2016), as well as academic and career retention, even when controlling for the personality trait conscientiousness (Eskreis-Winkler et al., 2014). Gritty people are more willing to expend effort to attain their selected goals (Silvia et al., 2013) and ignore less relevant goals (Duckworth and Gross, 2014).

Originally, Duckworth proposed Grit as a compound trait comprising of Consistency of Interest and Perseverance of Effort (Duckworth and Quinn, 2009). *Consistency of Interest* (*Consistency*) reflects the ability to focus on a small set of relevant goals related to the pursuit of the larger, more important objective. *Perseverance of Effort* (*Perseverance*) reflects effort toward one’s enduring or superordinate goal. Although the two facets of grit are correlated, recent research shows that they may be differentially related to behavioral outcomes associated with grittiness (Silvia et al., 2013; Bowman et al., 2015; Credé et al., 2017). For instance, Silvia et al. (2013) found that Grit–Perseverance was associated with increased effort on an anagram task whereas Grit–Consistency correlated negatively with physiological measures of effort, expended on the task. Thus, the authors concluded that individuals high on perseverance were more likely to appraise the anagram task as important and consequently expended more effort on it (Silvia et al., 2013).

Although predictive of academic success, grit was traditionally conceptualized as distinct from cognitive capacities, such as attention (Duckworth et al., 2007). However, the characterization of grit as the individual’s ability to maintain a highly valued long-term goal assumes the engagement and consumption of attentional resources. Empirical evidence supporting this assumption comes from a recent report of two experiments conducted by DiMenichi and Richmond (2015). DiMenichi and Richmond (2015) observed in their second experiment that individuals high on grit demonstrated better sustained attention than individuals low on grit. But, it’s critical to highlight that DiMenichi and Richmond (2015) did not assess their participants’ baseline grit levels using the Grit Scale (Duckworth et al., 2007). Instead they manipulated grit levels in their experiment by having their participants write about a time when they failed despite hard work (i.e., high grit condition) or a time when they succeeded after hard work (i.e., low grit condition) or the plot of a recent movie they watched (i.e., control condition). Hence, it’s possible that the observed effect DiMenichi and Richmond (2015) reported is driven by *state* rather than *trait* characteristics of grit. In addition, the researchers did not assess differential effects of the two facets of grit despite the fact that the sample, in their first experiment (wherein individual differences attention was not assessed), had significantly higher scores on Grit–Perseverance than Grit–Consistency (DiMenichi and Richmond, 2015).

Since gritty individuals are presented as persistently pursuing their goals by ignoring obstacles, setbacks, and distractions (Duckworth and Gross, 2014), it also is plausible to speculate that grit may be related to individual differences in attention control capacities. Duckworth and Gross (2014) claim that the gritty individuals may be particularly adept at ignoring or not developing irrelevant goals that conflict with their long-term passion. Thus, the observed success of gritty individuals may be due to their ability to focus their attention toward their highly valued superordinate goal. Recent work with resting state fMRI does provide some support for this notion. This line of research has shown that grit is associated with positive functional connectivity between the ventral striatal region and the medial prefrontal cortex (Myers et al., 2016). These regions are implicated in reward processing. The ventral striatum, in particular, is associated with predicting future rewards (Myers et al., 2016). In essence, these findings may indicate that grit is supported by networks of cognitive and emotional regulation that allow gritty individuals to maintain their focus on a long-term goal even when immediate rewards are intermittent or absent (Myers et al., 2016). However, it’s important to note that the researchers did not assess associations between neural correlates of grit and relevant cognitive capacities (i.e., attention).

Proponents of grit posit that high versus low levels of grit can account for differences between individuals in task performance (Credé et al., 2017). Less distracted by irrelevant goals and less discouraged by setbacks, individuals high on grit are better at capitalizing on opportunities and abilities than individuals low on grit (Duckworth and Gross, 2014). However, some studies have raised questions about this supposition (Maddi et al., 2012; Ivcevic and Brackett, 2014; Bazelais et al., 2016). For instance, grit predicted first year retention but not cadet performance at United States Military Academy (Maddi et al., 2012). In addition, empirical evidence indicates that highly gritty individuals are more likely to resist changing direction and strategies (Lucas et al., 2015), even when failing at a task. In a series of carefully controlled experiments, Lucas et al. (2015) showed that highly gritty individuals preferred to solve to difficult anagrams and persistently expended more effort in a game even when made aware of the fact that they were losing. The researchers speculated that the ability to ‘push through failure’ meant that highly gritty individuals were less responsive to feedback about their performance. Thus, it’s possible that gritty persistence without adequate attentional control may lead to failure on the task. Unfortunately, the relations between grit and individual differences in attentional capacities remains underexplored.

Posner and Petersen (1990) and Posner (2008) have previously proposed that attention comprises of three neural networks that have different functions. The three networks are: (1) Alerting – achieving and maintaining sensitivity toward incoming information, (2) Orienting – focus on select information from ongoing sensory inputs, and (3) Executive Control – resolving the conflict amongst possible responses (Fan et al., 2002). In a more recent review, Petersen and Posner (2012) found that their basic framework has been mostly validated by empirical work, with some need for elaboration. For instance, two forms of alerting have been distinguished in recent studies: tonic, a sustained form of vigilance over a long period of time (across blocks throughout a task), and phasic, a time limited enhancement produced by a warning single on a single trial time frame. Tonic alertness is an endogenous top down control process that is characterized by self-initiated preparedness to respond to incoming information (Posner, 2008; Sadaghiani and D’Esposito, 2015). Phasic alertness, on the other hand, is externally driven and characterized by selective processing of particular aspects of the incoming stimulus (Sadaghiani and D’Esposito, 2015). In addition, the orienting network and the executive network may actually each consist of two separable networks. The orienting network consists of a bilateral dorsal top-down control system and a ventral bottom-up orienting system. With respect to the executive network, initiation of a task seems to draw on a network of areas in lateral prefrontal cortex and parietal cortex (Petersen and Posner, 2012).

The measurement of these three distinct networks, identified by Posner and Petersen (1990), can be conducted behaviorally using the Attention Network Test (ANT; Fan et al., 2002). Over the last decade or so the ANT task has been successfully used to demonstrate abnormalities in attentional mechanisms of individuals with borderline personality disorder (Posner et al., 2002), trait anxiety (Pacheco-Unguetti et al., 2010), and extrinsic motivation (Robinson et al., 2012). Hence, we used the ANT (Fan et al., 2002) task to assess individual differences in attentional capacities for the two reported studies.

We present the results of two studies designed to examine associations between attention networks and the two facets of Grit: Consistency and Perseverance. Study 1 is a behavioral examination of the relations between grit and attention networks. In Study 2, we assessed neural correlates associated with performance on the ANT (Neuhaus et al., 2010) and their relation to the facets of Grit–Perseverance of Effort and Consistency of Interest. A number of recent studies have examined event related potentials produced during the ANT (Neuhaus et al., 2010; Galvao-Carmona et al., 2014). For example, Neuhaus et al. (2010) identified a post-target N1 effect that reflected differences due to cue. For posterior electrodes, the N1 had the largest negative amplitude for the spatial cue, then for the double cue, followed by the central and no cue whose N1 amplitude post target did not differ. They identified the difference in mean amplitude between the double cue ERP and the no cue ERP (aggregated over parietal electrodes Pz, P3, P4, PO9, PO10, O1, and O2) as the *alerting ERP effect*; the mean amplitude difference between the spatial cue ERP and center cue ERP as the *orienting ERP effect* (aggregated over the same electrodes). Neuhaus et al. (2010) also examined the effects on midline ERPs post target of the flanker condition as a measure of the neural correlate of inhibition. They found a reduction in the amplitude of the P3 component on Pz due to the added demands of inhibiting incongruent flankers.

DiMenichi and Richmond (2015) reported that highly gritty individuals also showed better sustained attention, therefore our first prediction was that performance on the ANT (Fan et al., 2002) would be related to individuals’ scores on the Grit Scale. Duckworth and Gross (2014) characterize gritty individuals as consistently engaged and working toward their superordinate goal, thus it is possible that gritty individuals may maintain a vigilant state of awareness. In which case, individuals who had high grit scores would also likely exhibit improved performance on the ANT (i.e., higher accuracy scores and shorter reaction times). However, there is also evidence showing that gritty individuals resist changing direction or strategies (Lucas et al., 2015). Since, Posner and Petersen (1990) have demonstrated that allocating attention to new information includes shifting away or disengaging from the current focus of attention, it is possible that individuals with high grit may perform worse on the ANT (i.e., lower accuracy scores and longer reaction times). Since past research has demonstrated that Grit–Perseverance and Grit–Consistency are related but distinct facets of grit (Duckworth and Quinn, 2009), we were interested in examining the relation between the two facets of grit and performance on the ANT. Some studies shown that Grit–Perseverance is the facet of grit that is likely to be correlated with cognitive and academic performance (Bowman et al., 2015; Credé et al., 2017). Thus, our second prediction was that participants’ performance on the ANT would be associated with Grit–Perseverance, not Grit–Consistency. As this is the first report, to the best of our knowledge, of a relation between grit and attention networks we did not make specific predictions about the direction of the relation and considered it an empirical question. Based on the results of the behavioral examination of the relations between Grit–Perseverance and ANT in Study 1, our primary prediction for Study 2 was that difference in mean amplitude of N1 between the double cue ERP and the no cue ERP (i.e., *alerting effect*) will be positively correlated with Grit–Perseverance. Prior research has indicated that the alerting is associated with activation in the frontal and parietal areas (Fan et al., 2005) and an enhancement of N1 amplitude (Neuhaus et al., 2010). Considering that our primary interest was in examining the relation between the alerting effect and grit and guided by previous electrophysiological findings with the ANT (Neuhaus et al., 2010), we chose to focus on the N1 component.

## Materials and Methods

### Study 1 Participants

One hundred and thirteen college-aged (18–25 years; M*_age_* = 18.62) individuals (females = 78) completed the Grit Scale (Duckworth et al., 2007), the ANT (Fan et al., 2002) and a questionnaire requesting demographic information. In exchange for their participation in the study, individuals were given partial course credit. Individuals were recruited for the study until we reached the maximum number of participants approved by the institutional review board for this project. Majority of the participants were White (3% identified as African-American, 3% as Asian or Asian-American, 3% as Hispanic, and 1.5% as Native Hawaiian or Pacific Islander).

### Study 1 Measures

#### Attention Network Test

**FIGURE 1:**
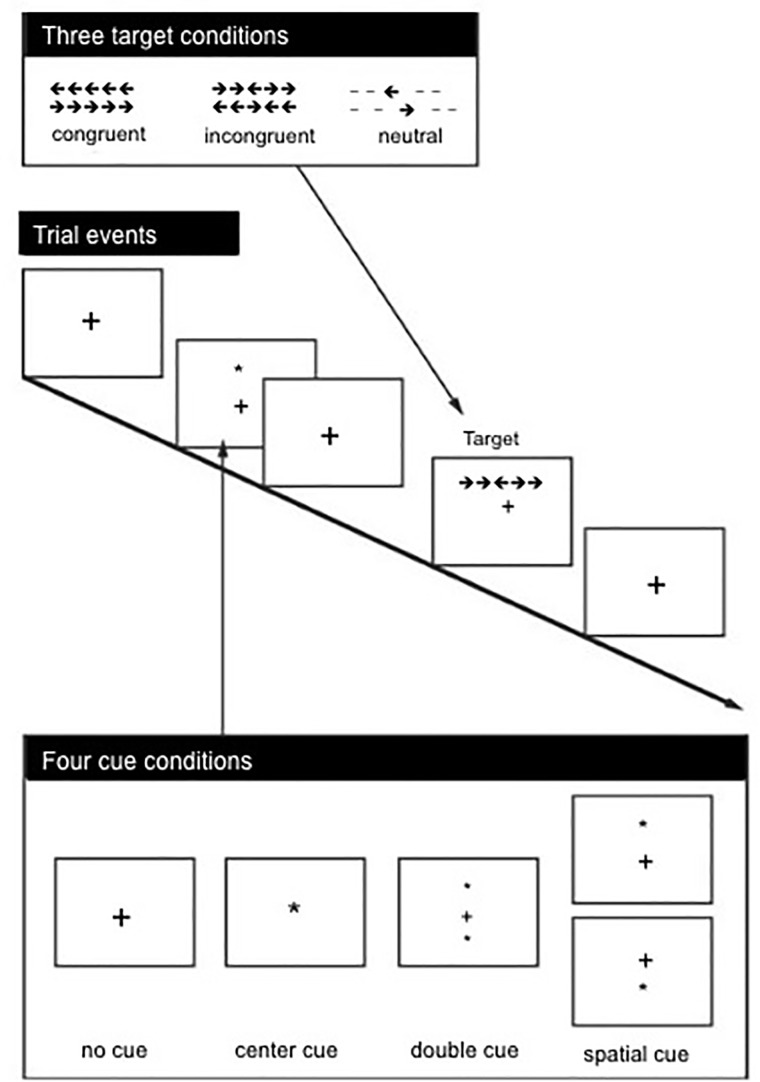
Attention Network Test timeline of a trial (Posner, 2008). Four types of cues presented none, double, central, or spatial (as shown). Target stimulus contains center arrow flanked by congruent arrows, incongruent arrows (as shown) or non-arrow lines. The Attention Network Scores are calculated using the following formulae: Alerting = RT no cue – RT double cue; Orienting = RT central cue – RT spatial cue; Executive Control = RT incongruent – RT congruent.

Attention Network Test (Fan et al., 2002): Attention networks were assessed using the ANT, which combines elements of the Posner cuing paradigm with the Ericksen flanker task. The fundamental task of the participant is to determine whether a central arrow is pointing to the left or right (Fan et al., 2002). The basic procedure for the ANT is schematized in **Figure 1**.

On a trial of the ANT, a fixation cross appears and remains in the center of the display. After a variable interval (range 400–1600 ms), a cue (an asterisk) appears if the trial were in one of the cue conditions: double cue, central cue, spatial cue. In the double cue condition, two asterisk cues appear both above and below the fixation cross. In the central cue condition, the asterisk replaces the fixation cross. In the spatial cue condition, the asterisk appears either above or below the fixation cross and is 100% valid in indicating where the target stimulus will appear. The cue, when present, remains on for 100 ms followed by a 400 ms cue-target interval at the conclusion of which the target appears.

The target stimulus is a collection of lines or arrows organized horizontally with the specific selection of characters depending on the target condition: neutral, congruent, or incongruent. In the neutral target condition, a center arrow is flanked by horizontal lines. In the congruent target condition, the center arrow is flanked by identical arrows pointing the same direction. Finally, in the incongruent target condition, the central arrow is flanked by arrows pointing in the opposite direction. The participant’s task is to focus attention on the central arrow and indicate by pressing the left key on a four button response box if the central arrow points left, and pressing the right key if the central arrow pointed right. Participants were instructed to use their left hand for the left response and their right hand for the right response. In addition, the participants were instructed to achieve high accuracy but to respond as quickly as possible. Following a practice session of 24 trials that included feedback, three blocks of trials occurred with 96 trials of each type of cue and target condition counterbalanced across the blocks. During the experimental blocks, no feedback regarding accuracy was provided.

Per Fan et al. (2002), attention network efficiencies are calculated from difference scores of response times: alerting = mean correct RT (no cue condition) – mean correct RT (double cue condition); orienting = mean correct RT (center cue condition) – mean correct RT (spatial cue condition); conflict (executive control) = mean correct RT (incongruent condition) – mean correct RT (congruent condition).

#### Grit Questionnaire

The Grit Questionnaire (Duckworth et al., 2007) consisted of twelve items (e.g., *setbacks don’t discourage me*) on 5-point scales that range from *not like me at all* to *very much like me*. Items were reverse scored so that higher scores indicated that participants had relatively more grit. Based on Duckworth and Quinn (2009) we split the items into two subscales that correlated well with Grit: *Consistency of Interest* (α = 0.90) and *Perseverance of Effort* (α = 0.83).

### Study 1 Procedure

All procedures for the study were approved by the institutional review board. After giving informed consent, participants completed the ANT (Fan et al., 2002) on a computer, and then responded to the measure of grit and demographics on pen and paper.

### Study 2 Participants

Twenty-eight college-aged (18–21 years) individuals (females = 16) completed questionnaires for Grit (Duckworth et al., 2007) and demographics. All participants were right handed and were given course credit or received a $15 gift card. One participant did not complete the Grit Scale. Participants were recruited until we reached the maximum number of participants for the project approved by the institutional review board.

### Study 2 Measures and Procedure

Materials and measures were the same as Study 1 for the behavioral task, the demographics questionnaire and the Grit Scale (Duckworth et al., 2007). Based on Duckworth and Quinn (2009) we split the items into two subscales that correlated well with Grit: *Consistency of Interest* (α = 0.86) and *Perseverance of Effort* (α = 0.79). Concurrent with the behavioral performance on the ANT task, EEG was recorded.

Prior to their arrival to the EEG laboratory, they were asked to review a video that provided an overview of what to expect when participating in an EEG experiment. This video is part of the informed consent process for all studies using the EEG laboratory. EEG was recorded using the Net Amps 300 system from Electrical Geodesics, Inc. (EGI, Eugene, OR, United States) with 256 channel geodesic sensor nets (Tucker, 1993) and Netstation 4.5.6 software. Prior to recording, electrode impedances were brought down below 50 kΩ which is appropriate for high impedance systems per manufacturer guidelines. EEG data were sampled at 250 Hz referenced to Cz during recording. EEG was band-passed filtered 0.1–48 Hz offline prior to segmentation. For the construction of ERPs for the different cue conditions, EEG was segmented from 800 ms before target onset to 1,000 ms post target into the categories of No Cue, Double Cue, Spatial Cue, and Center Cue (collapsing across target flanker conditions) using only correct trials locked to target onset. Segments containing eye blinks, eye movements and too many bad channels (in excess of 10% of the total) were marked as bad and discarded. Bad channels were replaced using interpolation. Segments were baseline corrected using the interval 800–600 ms prior to target onset and then averaged to create the ERP and rereferenced to the electrode average using a montage operation. ERPs were created using at least 30 artifact free trials for all conditions. All analyses were performed using Netstation software, Ver. 4.5.6.

**FIGURE 2:**
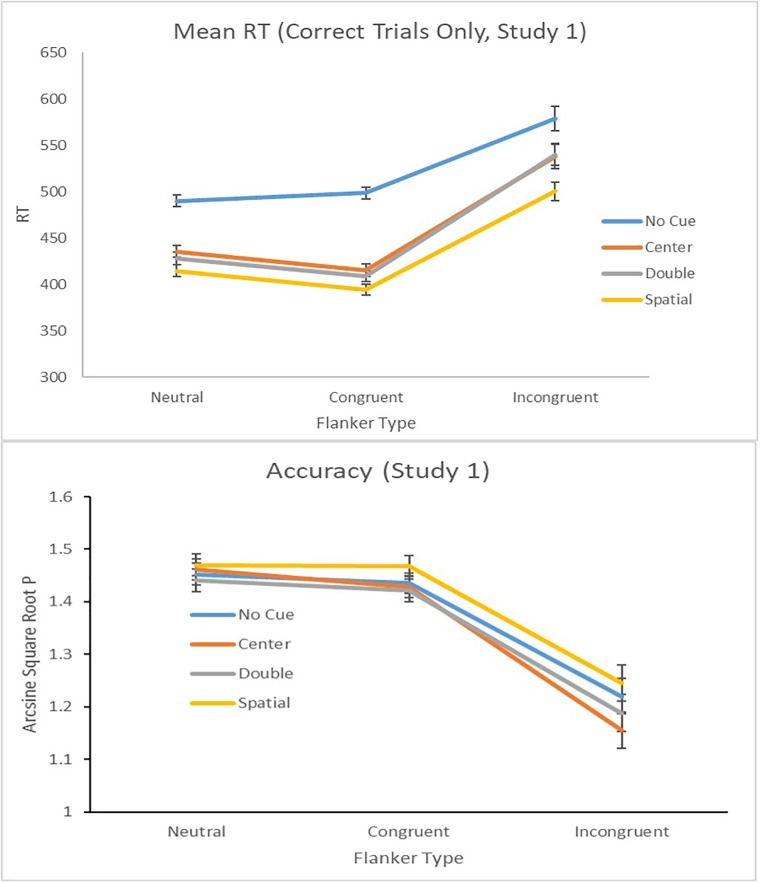
Mean response times with standard errors **(Top)** and (transformed) proportion correct **(Bottom)** as a function of cue and flanker conditions in Study 1.

**FIGURE 3:**
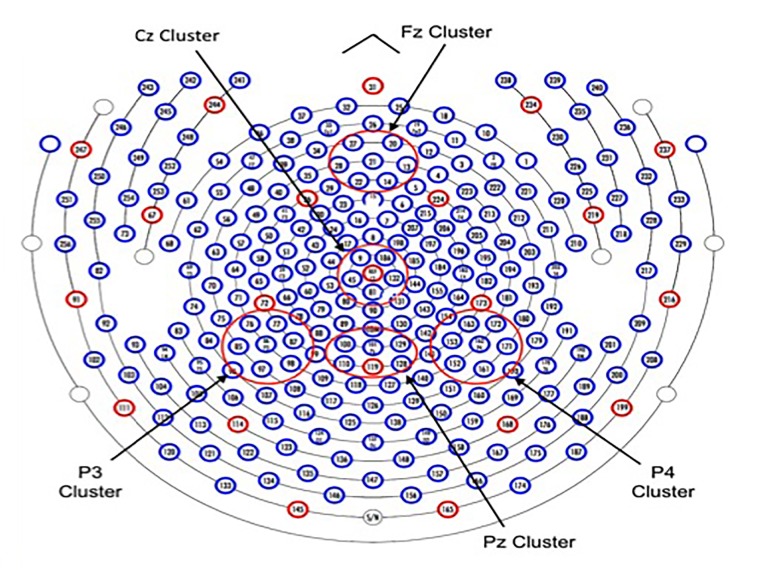
Schematic of the Sensor Net 256 Channel.

For ERPs indexing the flanker conditions, all steps in processing were the same except trials were grouped into neutral, congruent, and incongruent flanker categories (collapsed across cue conditions). Correct trials only were included. ERP effects associated with alerting, orienting, and inhibition were computed as differences in mean amplitudes across cue (for alerting and orienting) or flanker conditions. For the cue conditions, mean amplitude for each electrode in a cluster around P3, Pz, P4 was computed using a measurement window extending from 150 to 250 ms post target (**Figure 2**). *Alerting* is defined as the difference in mean amplitude between the double cue and the no cue conditions averaged across sensors in the respective clusters within this window. If the double cue produced a more negative N1 (as in Neuhaus et al., 2010) than the no cue condition as would be predicted by an efficacious alerting network, the result would be a negative mean amplitude alerting effect (this is important to understand the valence of the correlations with Grit). *Orienting* is defined as the difference in mean amplitude in the measurement window between the spatial and center cue conditions averaged across channels within the cluster. For the inhibition effect, mean amplitude was computed using a time window 350–500 ms post target for each of the flanker conditions and sensor within each of three clusters around Fz, Cz, and Pz. *Inhibition* is the difference in mean amplitude between the congruent and incongruent flanker conditions averaged across channels in the cluster. Note that the P3 amplitude is reduced for incongruent flankers relative to congruent for posterior electrodes (Neuhaus et al., 2010). Smaller differences are consistent with good cognitive control. **Figure 3** shows the electrodes in each of the studied clusters for the 256 geodesic sensor nets that were used.

## Results and Discussion

### Study 1

**Table 1** contains the means and standard deviations for accuracy (across congruent and incongruent flanker conditions and overall) in the Attention Network Task, as well as means and standard deviations for reaction time effects for Alerting, Orienting, and Executive attention subcomponents (Note: Nine participants were removed from the analysis due to zero accuracy in at least one condition). Participants RT data for correct trials were subjected to repeated measures ANOVA [Cue Level (4) × Flanker level (3)]. Mauchly’s test of sphericity emerged significant for Flanker level effects, and Cue^∗^Flanker interaction effects, therefore Greenhouse–Geisser corrected values were used for the analyses.

**Table 1:** Descriptive statistics of the behavioral measures of grit and performance on the Attention Network Test.

Variable	Study 1 (*N* = 104) *M (SD)*	Study 2 (*N* = 27) *M (SD)*
**Attention Network Test**		
Overall accuracy (percent)	90.47 (14.07)	96.10 (3.28)
Accuracy congruent (percent)	91.76 (10.94)	98.87 (2.02)
Accuracy incongruent (percent)	77.72 (24.35)	91.14 (7.04)
Alerting RT	62.87 (32.25)	48.55 (18.97)
Orienting RT	23.68 (28.10)	52.97 (25.36)
Conflicting RT	96.69 (63.59)	108.20 (30.64)
Overall RT	472.02 (78.28)	465.69 (188.06)
**Grit Scale**		
Overall	3.32 (0.55)	3.37 (0.49)
Perseverance of effort	3.86 (0.56)	3.76 (0.57)
Consistency of interest	2.79 (0.81)	2.97 (0.75)


The analysis indicated that the results for RT and accuracy mirrored typical behavioral findings on the ANT (Fan et al., 2002). The double cue trials produced reliably smaller RTs than no cue trials whereas the no cue trials had greater accuracy than double cue trials (Alerting; *ps* < 0.05). The spatial cue trials produced reliably smaller RTs and more accuracy than center cue trials (Orienting; *ps* < 0.05). Finally, the congruent flanker trials produced reliably faster RTs and more accuracy than incongruent flanker trials (Executive; *ps* < 0.05). The analysis for accuracy did not yield a significant Cue by Flanker interaction [*F*(5.48, 553.23) = 0.04, *p* = ns] however, there was a significant interaction effect for RT [*F*(2.97, 300.27) = 4.66, *p* < 0.01] (**Figure 2**).

Next, we conducted bivariate correlations between performance on the ANT and Girt–Consistency of Interest and Perseverance of Effort. See **Table 2** for details. RTs for the *alerting, orienting*, and *executive control* were positively correlated indicating that individuals had smaller RTs in one network were also more likely to be faster in another aspect of attention. Although the original report on the ANT demonstrated independence between the three networks (Fan et al., 2002), numerous studies since then have observed the relation between the networks of attention (Fuentes and Campoy, 2008).

**Table 2:** Bivariate correlations between perseverance of effort, consistency of interest and performance on the Attention Network Test.

Variable	Grit-PE	Grit-CI	Alerting	Orienting	EC	Accuracy	Accuracy congruent	Accuracy incongruent
**Study 1 (*N* = 104)**								
Grit-CI	0.27^**^	–						
Alerting	-0.20^*^	-0.10	–					
Orienting	-0.15	0.14	0.26^**^	–				
Executive control	-0.03	0.05	0.44^***^	0.13	–			
Percent accuracy	0.21^*^	-0.003	-0.03	-0.04	0.08	–		
Accuracy congruent	0.21^*^	0.02	-0.004	-0.12	0.25^*^	0.91^***^	–	
Accuracy incongruent	0.15	-0.04	-0.04	0.09	-0.16	0.86^***^	0.56^***^	–
Overall RT	-0.16	0.05	-0.12	-0.04	0.11	-0.44^***^	-0.45^***^	-0.32^**^
**Study 2 (*N* = 27)**								
Grit-CI	0.11	–						
Alerting	-0.35^+^	-0.11	–					
Orienting	-0.25	-0.25	0.43^*^	–				
EC	0.24	0.16	0.22	0.14	–			
Percent accuracy	-0.21	-0.26	0.04	0.33	-0.29	-		
Accuracy congruent	-0.10	-0.23	-0.20	0.11	-0.25	0.80^***^	-	-
Accuracy incongruent	-0.29	-0.19	0.15	0.37	-0.24	0.96^***^	0.63^***^	0.63^***^
Overall RT	-0.06	0.07	0.30	0.36	0.34	-0.25	-0.54^**^	0.04


*^+^p = 0.074; ^∗^p < 0.05, ^∗∗^p < 0.01; ^∗∗∗^p < 0.001. Grit-CI, Grit–Consistency; Grit-PE, Grit–Perseverance; EC, executive control.*

As we had predicted we found that Grit–Consistency was not correlated with participants’ performance on the ANT. This finding provides additional support for previous research showing that Grit–Perseverance not Consistency is associated with cognitive indicators of performance (e.g., Bowman et al., 2015) and indicates that Grit–Perseverance may be the more important facet of grit (Credé et al., 2017).

Our primary prediction was supported, Grit–Perseverance was associated with performance on the ANT. Specifically, we found that Grit–Perseverance was negatively associated with the *alerting effect* on the ANT (subtracting RT double-cue trials from no-cue trials). Although the strength of the correlation is modest, it is consistent with past research examining the relations between grit and cognitive variables (e.g., Bowman et al., 2015; Credé et al., 2017). In other words, we found that individuals high on Grit–Perseverance were likely to exhibit a smaller alerting effect. The *alerting effect* on the ANT refers to the individual’s ability to react to a warning signal in the visual field by enhancing their response preparation (Neuhaus et al., 2010). In timed tasks, it is expected that an individual’s response time would improve after a warning has been presented (Posner, 2008). In essence, the warning initiates a homeostatic change (Sadaghiani and D’Esposito, 2015) and individuals should be faster to respond in double cue trials in comparison to the no-cue trials (Posner, 2008). Examination of the mean RTs for the correct no cue (*M* = 522.96, *SD* = 83.08) and the correct double cue (*M* = 460.09, *SD* = 84.71) trials indicate this was case. However, when the RTs of the double cue trials (i.e., smaller RTs) were subtracted from the no cue trials (i.e., bigger RTs), individuals with high scores on Grit–Perseverance were likely to show a smaller mean difference in response time. Thus, our results indicate that gritty individuals have an attenuated alerting effect, as defined by the ANT.

There could be two explanations for this result. First, this might indicate that individuals with high Grit–Perseverance were less receptive to rapid changes in their attention field. This could be due to impairment in using the cue to enhance their response. Second, it may be gritty individuals are able to maintain alertness without the help of a cue (Urbanek et al., 2010). Based on Urbanek et al. (2010) we examined associations between Grit–Perseverance and mean correct RTs in the no cue condition and mean correct RTs in the double cue condition, which revealed that Grit–Perseverance was negatively associated with mean correct RTs in the no cue condition (*r* = -0.30, *p* = 0.002). However, there was no correlation between Grit–Perseverance and RTs in the double cue condition. In addition to RT, we checked for differences in accuracy in the no cue versus double cue condition as a function of High (*M* = 4.36, *SD* = 0.33, *n* = 48) versus Low (*M* = 3.45, *SD* = 0.37, *n* = 56) Grit–Perseverance. A 2 × 2 mixed ANOVA, with Condition (No cue vs. Double cue) as the within subjects factor and Grit (High vs. Low) as the between subject factor was conducted. The analysis revealed a significant main effect [*F*(1, 102) = 6.63, *p* = 0.01, η_p_^2^ = 0.06] which was qualified by a significant interaction effect [*F*(1, 102) = 6.32, *p* = 0.01, η_p_^2^ = 0.06]. Participants were more accurate in the no cue condition (*M* = 1.34, *SD* = 0.26; arc sine transformed) in comparison to the double cue condition (*M* = 1.32, *SD* = 0.26; arc sine transformed). However, individuals with high Grit–Perseverance were less accurate in the double cue condition (*M* = 1.30, *SD* = 0.25; arc sine transformed) than in the no cue condition (*M* = 1.34, *SD* = 0.26; arc sine transformed). For individuals with low Grit–Perseverance there was do difference in accuracy in the no cue (*M* = 1.34, *SD* = 0.27; arc sine transformed) versus double cue conditions (*M* = 1.34, *SD* = 0.27; arc sine transformed).

The double cue condition is supposed to evoke preparedness in the individual by warning them about an impending target, which should yield a faster response (Posner, 2008). Individuals who were high on Grit–Perseverance were likely to be faster in the no cue conditions but their performance in the double cue condition was unrelated to their grit levels. This could indicate that although all the participants responded equivalently to the warning cue, highly gritty individuals were more likely to exhibit shorter RT in the no cue trials. In which case, our findings provide support for the notion that highly gritty individuals exhibit better sustained attention than individuals low on grit (DiMenichi and Richmond, 2015). However, we also found that individuals with high Grit–Perseverance were less accurate in the double cue trials, essentially indicating that the cues were less effective as a warning signal. Duckworth believes that gritty individuals are more likely to be intrinsically motivated (Duckworth et al., 2007), so it is possible that gritty individuals may have enhanced tonic alertness or vigilance. Considering that this is the first report of this association and that we did not experimentally manipulate grit or cue conditions systematically, caution should be exercised when interpreting and generalizing this finding. Its also important to point out that participants were at ceiling levels in accuracy, so it would be in appropriate to make any firm claims based on the accuracy data. Future studies should examine this effect by manipulating task difficulty, exposure time, and size of the cues on the ANT to explore associations with highly gritty individuals.

Previous research has shown that extrinsic motivation (i.e., monetary rewards) can have an impact on performance on the ANT (Robinson et al., 2012). Participants who were given an incentive demonstrated a significant improvement in their alerting network response times (Robinson et al., 2012). We were able to extend this work by examining relations between the trait-based ability to maintain a motivational state long-term and performance on the ANT. However, the direction of the relation between motivation and performance that we observed varies from, one found by, Robinson et al. (2012). Robinson et al. (2012) reported that participants in the externally motivated condition exhibited improved alerting, as compared to baseline levels. Specifically, the externally motivated group demonstrated reduced RT in the double cue condition only. In contrast, there was no change in the alerting RT of individuals in the control condition, as compared to baseline. In addition, for participants in the externally motivated condition higher scores in intrinsic motivation were associated with a ‘reduced cuing advantage’ (Robinson et al., 2012). Which could indicate that intrinsically motivated individuals are less responsive to cues within a context in which performance is associated with external rewards. Overall, the differences in our findings could be explained by the inhibitory effect of extrinsic rewards on intrinsic motivation demonstrated by Deci et al. (1999). When motivation is elicited through the potential for monetary reward, it heightens activity in brain circuits associated with impulsive and short-term rewards (Padmala and Pessoa, 2011). This means that individuals become more sensitive to reward-related cues. This sensitivity can enhance an individual’s preparatory cue-related activity on attentional tasks, like the ANT (Padmala and Pessoa, 2011). But, in individuals who are intrinsically interested and motivated, external cues and rewards can inhibit performance (Deci et al., 1999). Duckworth believes that gritty individuals are motivationally oriented and persist with their long-term, superordinate, goal by suppressing rival short-term and long-term goals that conflict with their passionate pursuit (Duckworth and Gross, 2014). Our finding, that high Grit–Perseverance is negatively correlated to the alerting effect, provides some support for this notion. However, it is also likely that individuals with high Grit–Perseverance exhibited ‘impulsive’ performance during the double cue trials. Although it is clear that individuals with high Grit–Perseverance showed better sustained attention, but faster response in no cue and less accuracy in double cue conditions could also be in an indicator of an ‘impulsive’ response. It’s important to note that the typical response pattern is faster RT and more errors in double cue conditions, which we observed with high on Grit–Perseverance, but not with those low on Grit–Perseverance. Thus, our data indicate differences in response patterns associated with the ANT under warning cue conditions. Future research that systematically accounts for external reward and feedback mechanisms on the ANT with highly gritty individuals will be able to address this issue.

### Study 2

**FIGURE 4:**
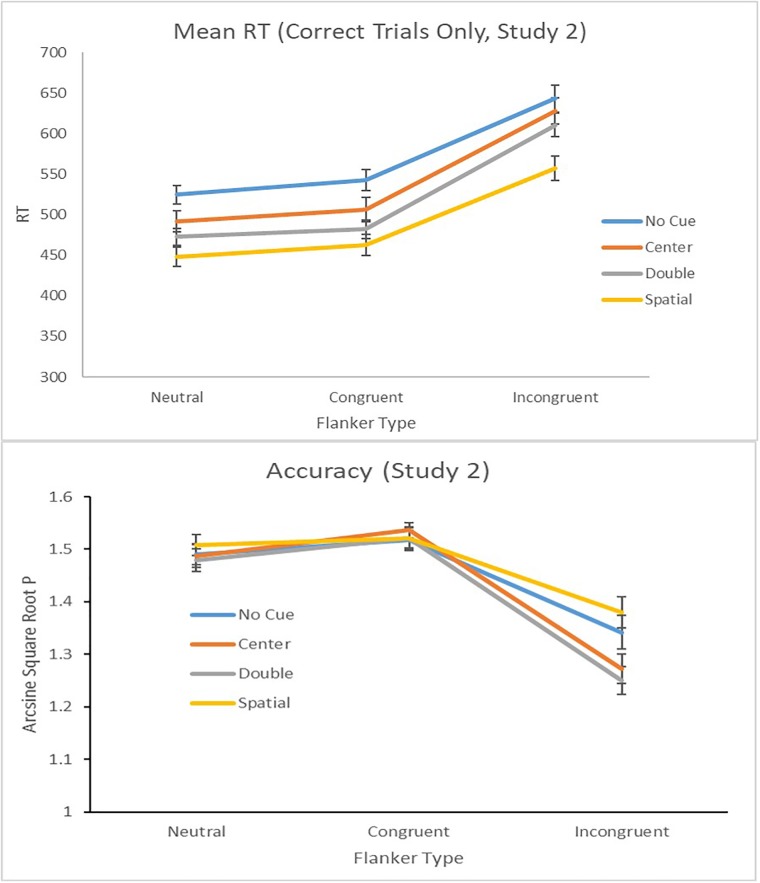
Mean response times with standard errors **(Top)** and (transformed) proportion correct **(Bottom)** as a function of cue and flanker conditions in Study 2.

**Tables 1, 3** contain means and standard deviations of all variables of interest. **Figure 4** presents the behavioral reaction times and accuracy scores on the ANT.

**Table 3:** Descriptive statistics of ERP components across conditions in the Attention Network Test (*N* = 27).

ERP	No cue	Double cue			Center cue	Spatial cue		
	*M (SD)*	*M (SD)*	*t*(27)	*p*	*M (SD)*	*M (SD)*	*t*(27)	*p*
N1 Pz	-0.47 (2.63)	-1.72 (2.54)	2.91	0.007^∗∗^	-0.61 (3.38)	-0.81 (3.72)	0.31	0.76
N1 P4	-0.99 (1.99)	-2.11 (2.29)	3.17	0.004^∗∗^	-0.94 (2.07)	-1.23 (2.86)	0.85	0.41
N1 P3	-1.74 (1.59)	-2.86 (1.99)	3.42	0.002^∗∗^	-1.86 (1.57)	-2.43 (2.26)	1.74	0.09
N1 O1 and O2	-1.69 (2.11)	-2.69 (1.89)	2.62	0.014^∗^	-1.43 (2.36)	-2.62 (2.71)	1.87	0.07
N1 Pooled	-1.22 (1.82)	-2.34 (1.81)	4.06	0^∗∗∗^	-1.21 (1.81)	-1.77 (2.54)	1.26	0.22

	**Congruent**	**Incongruent**						
	***M (SD)***	***M (SD)***	***t*(27)**	***p***				

Late Pos Pz	3.33 (4.06)	4.54 (0.86)	0.88	0.38				
Late Pos Fz	3.63 (0.69)	3.61 (0.68)	-2.61	0.014^∗^				


*^∗^p < 0.05, ^∗∗^p < 0.01; ^∗∗∗^p ≤ 0.001.*

#### Behavioral Performance on the ANT

We present the behavioral (RT and accuracy) results for the ANT task to ensure that the basic pattern of results typical of this task have been obtained with our sample. For response times, a four cue type by three flanker condition repeated measures ANOVA was conducted. Because Mauchly’s Test of Sphericity was rejected (all *p*s > 0.01), we present the Greenhouse–Geisser results. We found a significant effect of cue type, flanker condition, as well as a cue by flanker interaction (all *p*s < 0.01). Pairwise comparisons indicated that the results for RT and accuracy mirrored typical behavioral findings on the ANT (Fan et al., 2002). Due to the ceiling effects observed, we transformed accuracy data into proportion correct using arcsine of the square root of *p* (Fields, 2013). To be consistent and conservative, we present the Greenhouse–Geissler results. The analysis revealed a significant effect of cue type, flanker condition, as well as an interaction between cue and flanker types, (all *p*s < 0.01). These patterns in both RTs and accuracy replicate previously obtained results with the ANT (see, e.g., Neuhaus et al., 2010, 2011; Abundis-Gutierrez et al., 2014).

**Table 2** presents the results of bivariate correlations conducted to examine associations between behavioral performance on the ANT and Grit–Consistency and Perseverance. Consistent with Study 1, *alerting* (RT difference between double and no cue conditions) and *orienting* (RT difference between spatial and center cue conditions) were positively associated. Also, concordant with Study 1, we found individuals were faster to respond in double cue trials in comparison to the no-cue trials. Examination of the mean RTs for the correct no cue (*M* = 570.41, *SD* = 69.70) and the correct double cue (*M* = 521.85, *SD* = 63.27) trials indicate this was case. In addition, we found no association between Grit–Consistency and performance on the ANT. However, Grit–Perseverance was marginally associated with *alerting* (*r* = -0.35, *p* = 0.074), although the direction of the relation was the same as that reported in Study 1. Grit–Perseverance was not correlated with either mean correct RT in the double cue or no cue conditions (*p*s > 0.05). Similar to Study 1 we conducted a 2 × 2 mixed ANOVA, with Condition (No cue vs. Double cue) as the within subjects factor and Grit (High vs. Low) as the between subject factor was conducted. The analysis revealed no significant main effect or interaction effect (*p*s > 0.10). See **Table 3** for the descriptive statistics of the N1 ERP component across cue conditions. Overall the pattern of results observed in this study was concordant with the report by Neuhaus et al. (2010).

#### Neural Correlates of ANT and Perseverance of Effort

Recall that Neuhaus et al. (2010) found an enhanced negativity due to both alerting and orienting that occurred roughly 200 ms post target. They referred to this component as the target N1. They also identified an ERP correlate of the inhibition effect, a depression in the magnitude of a late positive component (extending 350–500 ms post target) for channel Pz.

Bivariate correlations were conducted between ERPs for alerting, orienting, executive control and scores on Grit–Consistency and Perseverance. As predicted, the analysis revealed a significant positive association between Grit–Perseverance and Pz *alerting*, and P3 *alerting* (**Table 4**). In essence individuals who had higher scores on Perseverance were more likely to demonstrate smaller mean difference in N1 amplitudes for double cue relative to no cue. Since the *alerting effect* is indexed by enhanced negativity of the N1 amplitude (Neuhaus et al., 2010), increased negativity indicates heightened alertness. Thus, a smaller mean difference in N1 amplitudes between double cue and no cue conditions signifies an attenuated *alerting effect*.

**FIGURE 5:**
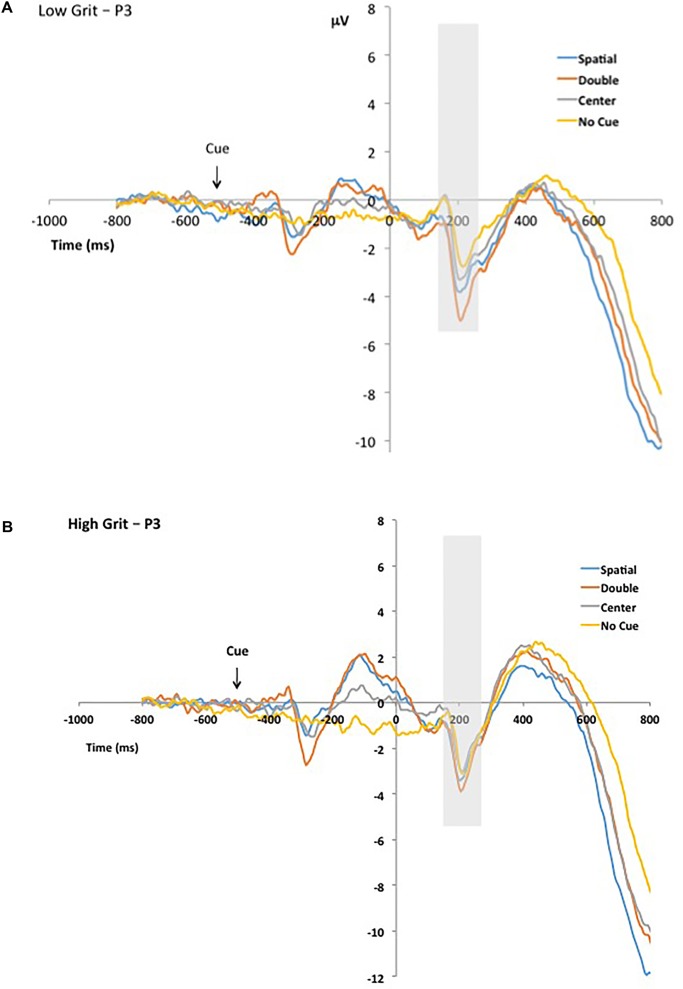
**(A)** ERPs for P3 cluster for those who scored low on the Grit Scale. Measurement window for ERP mean amplitude is shown as gray bar. **(B)** ERPs for P3 cluster for those who scored high on the Grit Scale. Measurement window for ERP mean amplitude is shown as gray bar.

**FIGURE 6:**
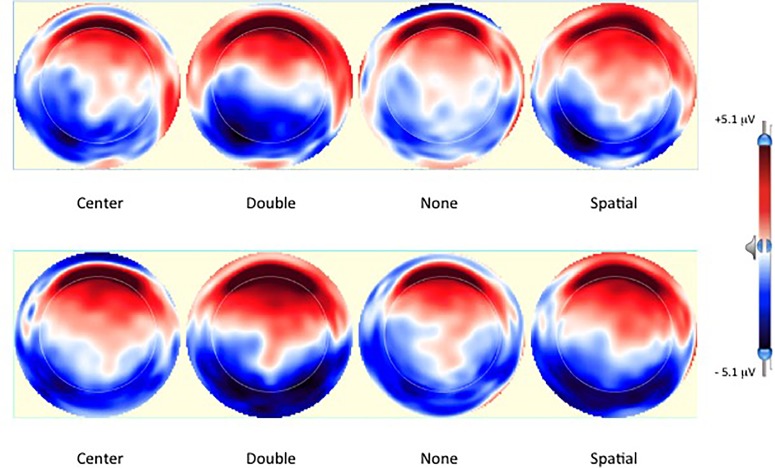
Topo maps of voltage 200 ms post target onset as a function of cue condition. **(Top)** Represents the low Grit group. **(Bottom)** Represents the high Grit group.

Using the mean Grit overall score we split the sample into low (*n* = 12; *M_grit_* = 2.94, *M_perseverance_* = 3.49, *M_consistency_* = 2.40) and high (*n* = 15; *M_grit_* = 3.71, *M_perseverance_* = 3.99, *M_consistency_* = 3.43) grit individuals. **Figure 5A**, graphs the ERPs for channel cluster P3 for those participants scoring ‘low’ on the Grit measure defined by median split. **Figure 5B**, shows ERPs for those scoring high on Grit. As is shown in the figures, individuals high on grit demonstrate a smaller difference in N1 negativity from the no cue to double cue conditions (5b: double cue = orange, no cue = yellow). Finally, **Figure 6** illustrates the topo maps of voltage taken at 200 ms post target as a function of cue type with the top row for the low Grit group and the bottom row for the high Grit group.

**Table 4:** Descriptive statistics of neural correlates and correlations with grit – perseverance of effort and consistency of interest (*N* = 27).

Neural measures	*M* (*SD*) mV	Grit–Perseverance Pearson *r*	Grit–Consistency Pearson *r*
**ERPs**			
P3 Alerting	-1.14 (1.76)	0.42^*^	0.17
P3 Orienting	-0.61 (1.74)	0.50^**^	-0.18
P4 Alerting	-1.12 (1.91)	0.08	-0.07
P4 Orienting	-0.29 (1.85)	0.26	-0.14
Pz Alerting	-1.28 (2.28)	0.39^*^	0.11
Pz Orienting	-0.24 (3.54)	0.28	-0.37
Pz Inhibition	0.47 (3.33)	0.06	-0.08
Fz Inhibition	-0.95 (1.72)	-0.15	-0.04


The fact that high Grit–Perseverance was positively associated with reduced negativity of the N1 amplitude demonstrates that our results for Study 2 are consistent with our findings in Study 1. Its been proposed that N1 enhancement is an indicator than the brain is orienting to and processing relevant sensory information in a preliminary way (Neuhaus et al., 2010). In their report on the ERP correlates of the ANT, Neuhaus et al. (2010) demonstrated a low N1 amplitude in the no cue condition and a bigger N1 amplitude in the double cue condition. Our results are consistent with their findings (**Table 3**). But, it’s important to note that we did not find a correlation between behavioral performance in the no cue or double cue trials and Grit–Perseverance. Alerting is a non-specific response that is outside flexible control (Neuhaus et al., 2010) and the alerting network is strongly correlated with activation in the thalamus (Fan et al., 2005). Thus, it is possible to speculate, in combination with the results from Study 1, that individuals with high Grit–Perseverance are able to maintain a level of alertness. Past research has shown that gritty individuals are less sensitive to feedback, particularly when they are failing at a task (Lucas et al., 2015). Considering that *alerting* is an indicator of the individual’s sensitivity and readiness for new information (Petersen and Posner, 2012) our findings may indicate that individuals high on Grit–Perseverance may be less sensitive to new information during a timed task. This could be one underlying explanation for gritty people being able to ignore irrelevant goals and maintain focus on their highly valued superordinate goal (Duckworth and Gross, 2014).

Grit–Perseverance was also associated with reduced N1 negativity in *orienting effect* in the P3 cluster. Before we present any interpretation of this finding, it is important to point out that spatial attention is considered to be right hemisphere dominant (Shulman et al., 2010), yet we observed an association between the *orienting effect* in the P3 cluster (in the left parietal cortex) and Grit–Perseverance. Hence, this result should be interpreted with extreme caution. In addition, Grit–Perseverance was not correlated with behavioral performance, for the orienting effect and center or spatial cue trials, on the ANT. Although it is possible that the correlation between Grit–Perseverance and N1 negativity, associated with the *orienting effect*, suggests that spatial cues may have been less effective as a signal for individuals high on Perseverance; the absence of a significant correlation with behavioral performance prevents us from drawing any firm conclusions. *Orienting effect* is a marker for the ability to select sensory information for focused attention (Petersen and Posner, 2012) and is influenced by the goals of the individual as well as characteristics of the cue (Egeth and Yantis, 1997). It may be that gritty individuals are perhaps less biased to orient their attention to changes in their visual field (Klein, 2000), but our data cannot provide any clear insight on this issue. Future research should examine whether this is due to reduced ‘inhibition of return’ effect (Posner et al., 1985).

It’s also pertinent to highlight that the sample size of this study was small which could have affected the associations we’ve reported. This is especially relevant for the relation between Perseverance and *orienting effect*, since we did not observe it in the behavioral data for either Study 1 or Study 2. Regardless the neural results allude to diminished attention shifting abilities in individuals who are high on Perseverance, and may provide additional support to previous research showing that gritty individuals are resistant to changing direction or strategies (Lucas et al., 2015).

## General Discussion and Conclusion

The relationship between motivation and attention has experienced a resurgence of interest in the last few years (Braver et al., 2014). Empirical studies have shown that motivationally salient stimuli are preferentially processed by the attentional systems (Pessoa, 2009). Despite the continued interest in Grit, as a variable that predicts academic success, few studies have examined relations between facets of Grit–Consistency and Perseverance and relevant cognitive variables (Credé et al., 2017). We conducted two studies to investigate the association between Grit–Consistency and Perseverance and individual differences in attention networks. Study 1 presented the results of a large sample behavioral study wherein a negative association between *alerting effect* and Grit–Perseverance was revealed. In essence, individuals with high Grit–Perseverance were more likely to exhibit a reduced alerting effect. Study 2 demonstrated that EEG neural correlates underlying *alerting* and *orienting* networks were also associated with Grit–Perseverance. Participants who where high on Grit–Perseverance were also more likely to show reduced N1 negativity in the P3 and Pz clusters for *alerting*. To the best of our knowledge this is the first set of studies to report this association behaviorally and through neural correlates.

Our findings indicate that having very high levels of Grit–Perseverance may be associated with an attenuated alerting effect. Alerting is calculated through the use of warning cues that indicate when, but not where, the stimulus will present (Posner, 2008). It’s relevant to note that the alerting effect is calculated by subtracting response times in the double cue condition from the no cue condition. Although Grit–Perseverance was associated with lower reaction times in the no cue trials, it was unrelated to performance in the double cue condition. Thus, the attenuated alerting effect could be due to two reasons. First, it may be that gritty individuals maintain alertness without the help of a cue (Urbanek et al., 2010). The fact that we found Grit–Perseverance was correlated with lower reaction times in the no cue trials in Study 1 provides some support for this notion. Reaction time can be viewed as the resolution of the uncertainty regarding the appearance of the stimulus (Bertelson, 1967). In the foreperiod the presentation of cues can provide instructions to prepare for a behavioral response (Meiran, 1996). Consequently, cues presented prior to the target stimulus can encourage faster reaction times. This is known as the warning effect (Bertelson, 1967). A key component of anticipating the response, to the stimulus, is achieving and maintaining a state of ‘full attention’ (Bertelson, 1967). Since individuals with high Grit–Perseverance were more likely to have an attenuated alerting effect and shorter reaction times in the no cue trials, we believe that they exhibited better sustained attention. We are not the first to demonstrate this effect for gritty individuals. Previous work by DiMenichi and Richmond (2015) has demonstrated that individuals high on Grit–Perseverance also exhibited enhanced sustained attention.

Although the results of our studies are concordant with the findings reported by DiMenichi and Richmond (2015), there are a couple of key differences between our work and theirs that should also be kept in mind. First, the observed effects of the study by DiMenichi and Richmond (2015) could be the consequence of state-like attributes of grit. They did not measure the trait grit using Duckworth’s scale (Duckworth et al., 2007), instead they manipulated grit levels by asking participants to write about a time when they tried hard and yet failed. It is possible that state and trait attributes of grit influence cognitive performance in varied ways. Certainly, the fact that some researchers have speculated that grit may only emerge as a relevant variable influencing performance under challenging circumstances (Lucas et al., 2015) provides some support for this idea. Second, the attention tasks that were used in the two studies present differential task demands. DiMenichi and Richmond (2015) did not use the ANT (Fan et al., 2002) to assess attentional capacities. For their work, DiMenichi and Richmond (2015) used a modified version of the Sustained Attention Response Task (SART; Robertson et al., 1997). Recent research indicates that the SART is susceptible to speed-accuracy tradeoffs and may not be an appropriate measure for mindless attention (Dillard et al., 2014). Hence, future research should examine state versus trait attributes of grit and their association to attentional capacities.

An alternative explanation, for our results, could be that highly gritty individuals may be less responsive to warning cues in their visual field that are designed to evoke activity that prepare the individual for changes in the attention field (Urbanek et al., 2010). Some support for this notion comes from the fact that performance in the double cue trials was unrelated to Grit–Perseverance in Study 1 and Study 2. Additionally, individuals high on Grit–Perseverance were less accurate in the double cue trials in comparison to the no cue trials in Study 1. Although, Grit–Perseverance was positively correlated with percent accuracy, which indicates that individuals with high Grit–Perseverance were also more likely to have higher accuracy rates on the ANT. This proposal is consistent with the work of Lucas et al. (2015) who demonstrated that highly gritty individuals were more likely to ignore feedback, warning them to quit when failing in a task. Our studies provide no information about the participants’ ability to disengage their attention based on cue. Thus, we cannot make any conclusive claims about the attention disengagement in gritty individuals. Especially since we provided no feedback based on performance during the task. Future research should examine whether those high on Perseverance may be less sensitive to changes in their attention field. Although this could be helpful if gritty individuals wanted to avoid distractions (Duckworth and Gross, 2014), the reduced sensitivity could also be a limitation in a domain where the tasks are not well defined and success is established by making rapid assessments, changes, or responses. Some have speculated that high grit may be associated with reduced flexibility (Lucas et al., 2015) and empirical evidence suggests grittier individuals are more likely to persist when failing, even if persistence on a task is costly (Lucas et al., 2015). It could be that grittiness may only foster success in well defined and difficult tasks that depend on sustained and deliberate practice to accomplish (e.g., success in spelling bees; Credé et al., 2017). Thus, future research that systematically examines the sensitivity of gritty individuals to warning cues and rewards is warranted. That line of research may be able to show that having very high levels of Grit–Perseverance could negatively influence one’s ability to disengage their attention based on external cues. This would increase the likelihood of engaging in dysfunctional behavior like depressive rumination (Koster et al., 2011) or persisting with a difficult problem rather than shifting attention to an easier one (Lucas et al., 2015).

Our work is correlational and cannot address questions about the causal nature of the relation between grit and attentional networks. There is some evidence to indicate that the psychometric properties of the ANT (Fan et al., 2002), particularly the alerting effect, are questionable and can complicate the interpretation of findings with healthy (MacLeod et al., 2010) and mentally ill (Hahn et al., 2011) individuals. Since our work is exploratory we cannot be sure that the observed correlations would hold up to multiple testing. Thus, a replication of this work with a larger sample and other measures of attention will be desirable before any firm conclusions are drawn. Another limitation of our work is that we did not manipulate state level motivation (e.g., Robinson et al., 2012) and differences in task demands (e.g., easy versus hard task; Lucas et al., 2015) that may have a role to play in the relation between grit and attentional networks. The role of task demands may be a particularly important consideration, since past research has shown that individuals high on Grit–Perseverance are more likely to expend effort on challenging tasks because grit enhances that value of goals (Silvia et al., 2013). Although our analysis of accuracy data did yield any clear patterns we believe this may have been due to ceiling effects in accuracy levels. Additionally, we did not systematically assess tonic alertness in our participants. Previous research has shown that tonic or intrinsic alertness and phasic alertness are distinct aspects of alerting (Posner, 2008; Sadaghiani and D’Esposito, 2015) and differentially related to executive processes (Weinbach and Henik, 2012). An alternative explanation for our results is that gritty individuals, in our studies, prioritized accuracy over time reaction time and consequently were slower to respond but were also more accurate. The positive correlation between accuracy and Grit–Perseverance provides some support for this notion (**Table 2**). Future research should examine whether tonic and phasic alertness are similarly related to grit. The generalizability of our findings is limited because both the studies, reported here, sampled college students. Nevertheless our work contributes to the literature on the relation between motivation and cognition, in two important ways. First, we provide further evidence of the role of motivational factors in influencing cognitive performance (Pessoa, 2009). Second, our examination of the neural correlates of ANT adds to the limited work on brain mechanisms associated with grit (Myers et al., 2016).

## Author Contributions

VK developed the study concept and design. KO collected all the data for Study 2. RT, AD, and VK conducted data analyses and interpretation. VK drafted the manuscript, and RT, AD, and KO provided essential additions, revisions, and feedback. All authors approved this version of the manuscript for submission.

## Conflict of Interest Statement

The authors declare that the research was conducted in the absence of any commercial or financial relationships that could be construed as a potential conflict of interest.
